# Digital biomarkers of cognitive function

**DOI:** 10.1038/s41746-018-0018-4

**Published:** 2018-03-28

**Authors:** Paul Dagum

**Affiliations:** Mindstrong Health, 248 Homer Street, Palo Alto, CA 94301 USA

**Keywords:** Predictive markers, Diagnostic markers

## Abstract

To identify digital biomarkers associated with cognitive function, we analyzed human–computer interaction from 7 days of smartphone use in 27 subjects (ages 18–34) who received a gold standard neuropsychological assessment. For several neuropsychological constructs (working memory, memory, executive function, language, and intelligence), we found a family of digital biomarkers that predicted test scores with high correlations (*p* < 10^−4^). These preliminary results suggest that passive measures from smartphone use could be a continuous ecological surrogate for laboratory-based neuropsychological assessment.

## Introduction

By comparison to the functional metrics available in other disciplines, conventional measures of neuropsychiatric disorders have several challenges. First, they are obtrusive, requiring a subject to break from their normal routine, dedicating time and often travel. Second, they are not ecological and require subjects to perform a task outside of the context of everyday behavior. Third, they are episodic and provide sparse snapshots of a patient only at the time of the assessment. Lastly, they are poorly scalable, taxing limited resources including space and trained staff.

In seeking objective and ecological measures of cognition, we attempted to develop a method to measure memory and executive function not in the laboratory but in the moment, day-to-day. We used human–computer interaction on smartphones to identify digital biomarkers that were correlated with neuropsychological performance.

## Results

**Fig. 1:**
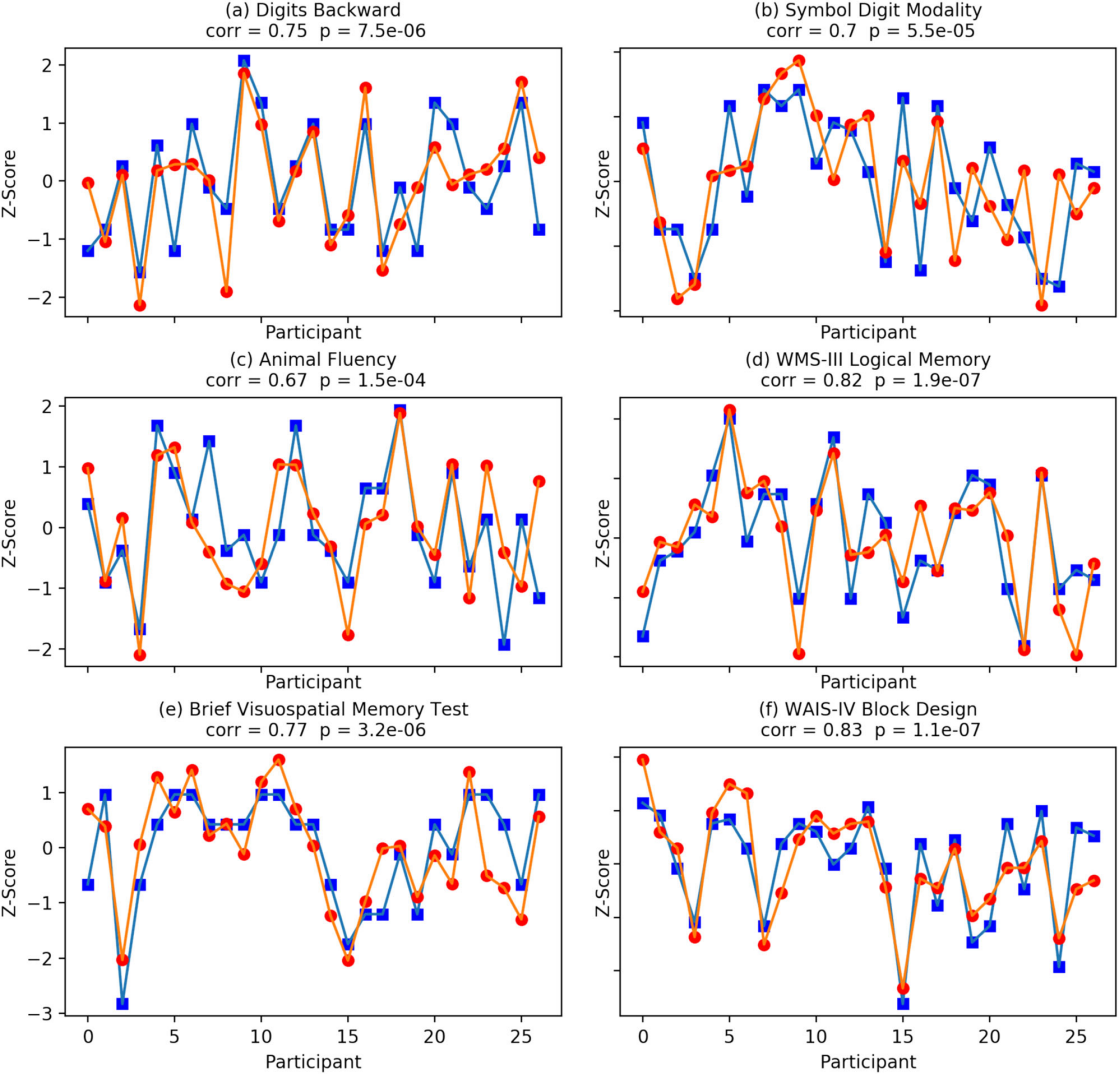
A blue square represents a participant test *Z*-score normed to the 27 participant scores and a red circle represents the digital biomarker prediction *Z*-score normed to the 27 predictions. Test scores and predictions shown are **a** digits backward, **b** symbol digit modality, **c** animal fluency, **d** Wechsler memory Scale-3rd Edition (WMS-III) logical memory (delayed free recall), **e** brief visuospatial memory test (delayed free recall), and **f** Wechsler adult intelligence scale-4th Edition (WAIS-IV) block design

In 2014, 27 participants (ages 27.1 ± 4.4 years, education 14.1 ± 2.3 years, M:F 8:19) volunteered for neuropsychological assessment and a test of the smartphone app. Smartphone human–computer interaction data from the 7 days following the neuropsychological assessment showed a range of correlations with the cognitive scores. Table 1 shows the correlation between each neurocognitive test and the cross-validated predictions of the supervised kernel PCA constructed from the biomarkers for that test. Figure 1 shows each participant test score and the digital biomarker prediction for (a) digits backward, (b) symbol digit modality, (c) animal fluency, (d) Wechsler Memory Scale-3rd Edition (WMS-III) logical memory (delayed free recall), (e) brief visuospatial memory test (delayed free recall), and (f) Wechsler Adult Intelligence Scale-4th Edition (WAIS-IV) block design. Construct validity of the predictions was determined using pattern matching that computed a correlation of 0.87 with *p* < 10^−59^ between the covariance matrix of the predictions and the covariance matrix of the tests.

**Table 1:** Fourteen neurocognitive assessments covering five cognitive domains and dexterity were performed by a neuropsychologist. Shown are the group mean and standard deviation, range of score, and the correlation between each test and the cross-validated prediction constructed from the digital biomarkers for that test

Cognitive predictions			
	Mean (SD)	Range	*R* (predicted), *p*-value
Working memory			
Digits forward	10.9 (2.7)	7–15	0.71 ± 0.10, 10^−4^
Digits backward	8.3 (2.7)	4–14	0.75 ± 0.08, 10^−5^
Executive function			
Trail A	23.0 (7.6)	12–39	0.70 ± 0.10, 10^−4^
Trail B	53.3 (13.1)	37–88	0.82 ± 0.06, 10^−6^
Symbol digit modality	55.8 (7.7)	43–67	0.70 ± 0.10, 10^−4^
Language			
Animal fluency	22.5 (3.8)	15–30	0.67 ± 0.11, 10^−4^
FAS phonemic fluency	42 (7.1)	27–52	0.63 ± 0.12, 10^−3^
Dexterity			
Grooved pegboard test (dominant hand)	62.7 (6.7)	51–75	0.73 ± 0.09, 10^−4^
Memory			
California verbal learning test (delayed free recall)	14.1 (1.9)	9–16	0.62 ± 0.12, 10^−3^
WMS-III logical memory (delayed free recall)	29.4 (6.2)	18–42	0.81 ± 0.07, 10^−6^
Brief visuospatial memory test (delayed free recall)	10.2 (1.8)	5–12	0.77 ± 0.08, 10^−5^
Intelligence scale			
WAIS-IV block design	46.1(12.8)	12–61	0.83 ± 0.06, 10^−6^
WAIS-IV matrix reasoning	22.1(3.3)	12–26	0.80 ± 0.07, 10^−6^
WAIS-IV vocabulary	40.6(4.0)	31–50	0.67 ± 0.11, 10^−4^

## Discussion

We have shown that we can generate digital biomarkers correlated with gold-standard neurocognitive tests using passively acquired data during daily use of a smartphone. Using supervised kernel PCA we can generate cross-validated predictions of the test scores with precision comparable to the gold-standard test–retest reliabilities.^1,2^ These digital biomarkers offer several advantages to conventional assessments. First, they are unobtrusive, placing no burden on the subject beyond the normal use of a smartphone. Second, they are ecological since the smartphone data is captured in a natural environment. Third, they provide dense daily assessments with potential insight into hour to hour or day to day variations in cognitive function. Lastly, they could scale globally with three billion smartphone users today, projected to 6 billion by 2020 (https://techcrunch.com/2015/06/02/6-1b-smartphone-users-globally-by-2020-overtaking-basic-fixed-phone-subscriptions/).

An obvious limitation of this pilot study is the small size (*n* = 27) relative to the large number of potential biomarkers (*n* = 1035). To counter the risk of over-fitting these results, predictions were made using leave-one-out cross validation (LOOCV), stringent confidence level (*p* < 10^−4^) and a simple linear kernel that was regularized. Nevertheless, these results should be considered preliminary until replicated in an independent sample. A further limitation is that the neuropsychological assessment occurred at one time point and the digital features were collected ecologically over the first 7 days following the assessment. For clinical assessments, one might argue that the real-world, continuous assessment would yield critical information relevant to function. Indeed, we postulate that the daily variability in the digital biomarkers will provide rich temporal insight into state-dependent changes in cognition and emotional health that may arise from disease and environmental effects. The selection of 7 days provided ecological data from which to select the peak value of each biomarker, which consistently led to the best predictions, suggesting that in the laboratory a participant performs at their best while in the real-world the participant’s function will deviate from peak depending on disease and environmental effects. Several large clinical studies will confirm our hypothesis and further establish the clinical utility of our approach.

## Method

All participants, recruited via social media, signed an informed consent form. Inclusion criteria required participants to be functional English speaking and active users of a smartphone. The protocol involved 3 h of psychometric assessment, installation of an app on their smartphone. The test battery is shown in the first column of Table 1. A single psychometrician performed all testing in a standard assessment clinic. The app on the phone ran passively in the background and captured tactile user activity that included swipes, taps, and keystroke events, collectively termed human–computer interactions (HCI).

From the HCI events we identified 45 event patterns. Each pattern represents a task that is repeated up to several hundred times per day by a user during normal use of their phone. Most patterns consisted of two successive events, such as tapping on the space-bar followed by the first character of a word, or tapping delete followed by another delete tap. Some patterns were collected in a specific context of use. For example, tapping on a character followed by another character could be collected at the beginning of a word, middle of a word, or end of a word. Each pattern generated a time-series composed of the time interval between patterns. The time-series were segmented into daily time-series. To each daily time-series we applied 23 mathematical transforms to produce 1035 distinct daily measurements that we term digital biomarkers.

For each participant we selected the first 7 days of data following their test date. A biomarker was considered a candidate for a neurocognitive test if over the 7 day window the 7 correlations between sorted biomarker values and the test scores were stable (meaning of the same sign). The two-dimensional design matrix for the supervised kernel PCA was constructed by selecting the peak value of each candidate biomarker over the 7 days. For each test, we constructed a linear reproducing Hilbert space kernel from the biomarkers and used a supervised kernel principal component analysis^3^ with LOOCV as follows. To predict the 1st participant test result the model fitting algorithm was run on the remaining participants without access to the 1st participant’s data, and so forth iterating 27 times to generate the 27 predictions.

### Code availability

The code that support the findings of this study are available on reasonable request from the author.

### Data availability

The data that support the findings of this study are available on reasonable request from the author.
